# β-Ionone for Cancer Therapy: Molecular Mechanisms, Apoptotic Signaling, Preclinical Evidence, and Translational Perspectives

**DOI:** 10.3390/ijms27219521

**Published:** 2026-10-24

**Authors:** Ajay Kumar, Pooja Sharma, Soniya Goyal, Shallu Saini, Damandeep Kaur, Gurpreet Kaur Bhatia, Sandeep Sharma, Hardeep Singh Tuli, Hajed Obaid Alharbi, Arshad Husain Rahmani

**Affiliations:** 1 Department of Environmental Science, Graphic Era (Deemed to Be University), Dehradun, Uttarakhand 248002, India; kumar.ajay1250@gmail.com; 2 Department of Bio-Sciences &Technology, Maharishi Markandeshwar (Deemed to Be University), Mullana-Ambala, Haryana 133207, India; 3 University Center for Research & Development (UCRD), Chandigarh University, Gharuan, Mohali, Punjab140413, India; 4 Department of Physics, MM Engineering College, Maharishi Markandeshwar (Deemed to Be University), Mullana, Ambala, Haryana 133207, India; 5 Centre for Research Impact and Outcome, Chitkara University, Rajpura, Punjab 140417, India; 6 Division of Research and Development, Lovely Professional University, Phagwara, Punjab 144411, India; 7 Centre of Excellence in Computational Research and Drug Discovery, Department of Bio-Sciences and Technology, Maharishi Markandeshwar Engineering College, Maharishi Markandeshwar (Deemed to Be University), Mullana, Ambala 133207, India; 8 Department of Medical Laboratories, College of Applied Medical Sciences, Qassim University, Buraydah 51452, Saudi Arabia

**Keywords:** Apoptosis, β-Ionone, cancer, Ferroptosis, natural compounds, Pharmacokinetics

## Abstract

β-Ionone is a naturally occurring C13-norisoprenoid apocarotenoid which is mainly derived from carotenoid cleavage and has gained significant attention due to its potential anticancer activity. Experimental investigations have shown that β-ionone has the ability to arrest the proliferation of cancer cells and to induce programmed cell death by several mechanisms depending on the type of tumors and experimental conditions. Mitochondrial apoptosis, such as Bax/Bcl-2 balance, cytochrome c release, activation of caspases, and p53-associated signaling, is the most consistently supported evidence for β-Ionone. Other mechanisms include modulation of the PI3K/Akt and MAPK/ERK signaling pathways, regulation of cell-cycle progression, generation of oxidative stress, and increased sensitivity to death-receptor-mediated apoptosis. Moreover, recent experimental studies have provided further support for the role of β-ionone in ferroptosis, as it was found to modulate the ferroptosis-associated proteins and Wnt/β-catenin signaling in gastric cancer models. By contrast, other mechanisms such as autophagy and the JAK/STAT3 signaling pathway are proposed mechanisms that need to be directly tested. Most importantly, evidence collected using β-ionone derivatives (such as esters and endoperoxides) differs from that collected from the parent. While promising preclinical results have been observed, translation is hampered by the lack of human pharmacokinetic data, uncertainties in bioavailability, and a lack of toxicity data and clinical trials. Target validation, characterization of the PHPD profile, safety assessment, formulation optimization, and clinically relevant in vivo validation should thus be given special attention in future studies.

## 1. Introduction

Cancer continues to pose a significant health threat in the world as it is growing in frequency and has increasing resistance to therapies, recurrence, and side effects of treatments [1]. According to GLOBOCAN data, there are millions of cancer cases each year, and prevalence and cancer-related deaths are projected to increase rapidly as a result of an ageing population, urbanisation, and lifestyle risk factors in modern society [2,3]. New developments in cancer treatments have significantly influenced the results of treatment for cancer patients; however, resistance to traditional therapies and the inability to remove malignant cells are still great challenges [4,5]. Natural sources rich in bioactive compounds have attracted increasing interest for their ability to inhibit numerous molecular targets that play a crucial role in the proliferation and survival of cancer cells, and have become the subject of substantial research as potential anticancer agents [6]. Among these compounds, β-ionone, a naturally occurring derivative of carotene degradation, has been shown to exhibit antiproliferative, proapoptotic, antioxidant, and chemopreventive properties, making it a promising molecule. β-Ionone is abundant in flowers, fruits, and vegetables and plays a key role in the aromatic properties of these plant parts [7]. It results from the oxidative breakdown of carotenoids such as β-carotene, primarily by carotenoid cleavage dioxygenases, and occurs naturally in grapes, berries, citrus, tomatoes, carrots, sweet potatoes, roses, violets, jasmine, and tea. Its various plant sources underscore its role as a volatile signalling molecule and its significance in plant biology beyond aroma [8]. In addition to its sensory properties, β-ionone has been shown to possess a wide range of biological functions, including antioxidant, anti-inflammatory, and anticancer activities [9,10,11]. Recent studies have reported that β-ionone inhibits tumor cell proliferation while inducing apoptosis in several cancer models by modifying mitochondrial function, survival signaling pathways, oxidative stress, and cell-cycle processes [9,10,11]. In addition to sensory and ecological uses, β-ionone has been found to possess pharmacological properties, owing to the ability of apocarotenoids derived from carotenoids to act on cellular signal transduction pathways associated with oxidative stress, inflammation, proliferation, and programmed cell death. Despite this, a thorough evaluation of cancer-specific analyses that integrate validated apoptotic mechanisms with novel cell death modalities and constraints remains limited. β-ionone and its derivatives have been shown to possess anticancer effects in several experimental models, but the exact molecular mechanisms underlying these effects remain poorly understood [12]. The deregulation of these apoptotic pathways is a characteristic feature of cancer and contributes to tumour progression and therapy resistance [13,14]. Thus, knowledge of the effects of β-ionone on apoptotic signalling, mitochondrial function, oxidative stress responses, and other cell death pathways is important for evaluating its potential anticancer activity. However, a cancer-specific analysis of β-ionone’s molecular mechanisms of inducing cell death is limited. This review offers a detailed evaluation of its anticancer effects, focusing on cancer models such as mitochondrial apoptosis, death receptor signaling, PI3K/Akt/mTOR and MAPK/ERK pathways, oxidative stress, ferroptosis, and therapeutic sensitization. It also distinguishes validated mechanisms from proposed ones and discusses pharmacokinetic limitations, safety, nanodelivery, and translational challenges for clinical use.

## 2. Methodology

The narrative review was also conducted by searching the PubMed, Scopus, Web of Science, and Google Scholar databases for relevant studies on β-ionone and apoptosis in cancer. The following keywords and combinations were searched: β-ionone, beta-ionone, apoptosis, cancer, cell cycle arrest, Bax, Bcl-2, caspase activation, p53, PI3K/Akt/mTOR, MAPK/ERK, JAK/STAT3, ROS, ROS generation, autophagy, natural compounds, and nanodelivery. A first search in the chosen databases yielded 120 records. Following the removal of duplicate records, titles, abstracts, and full-text articles were screened and deemed knowledge-defining for relevance to the mechanisms reviewed, resulting in 75 articles for inclusion in this review. The following types of articles were included: published articles, original research articles, mechanistic studies, in vivo preclinical cancer model studies, and relevant review articles. Articles that were not specifically focused on β-ionone activity but had implications for apoptotic mechanisms were chosen over other articles that did not directly report on β-ionone-induced apoptosis but were relevant to other aspects of its activity. Strict scientific criteria were used to select articles, and those lacking sufficient scientific detail or not mechanistically relevant, as well as duplicate and non-relevant studies, were excluded. The available literature was critically analysed, and the major molecular mechanisms, therapeutic applications, safety profile, and future translational applications of β-ionone in cancer treatment are summarised.

## 3. Chemistry, Structural Activity and Pharmacokinetics

**Figure 1:**
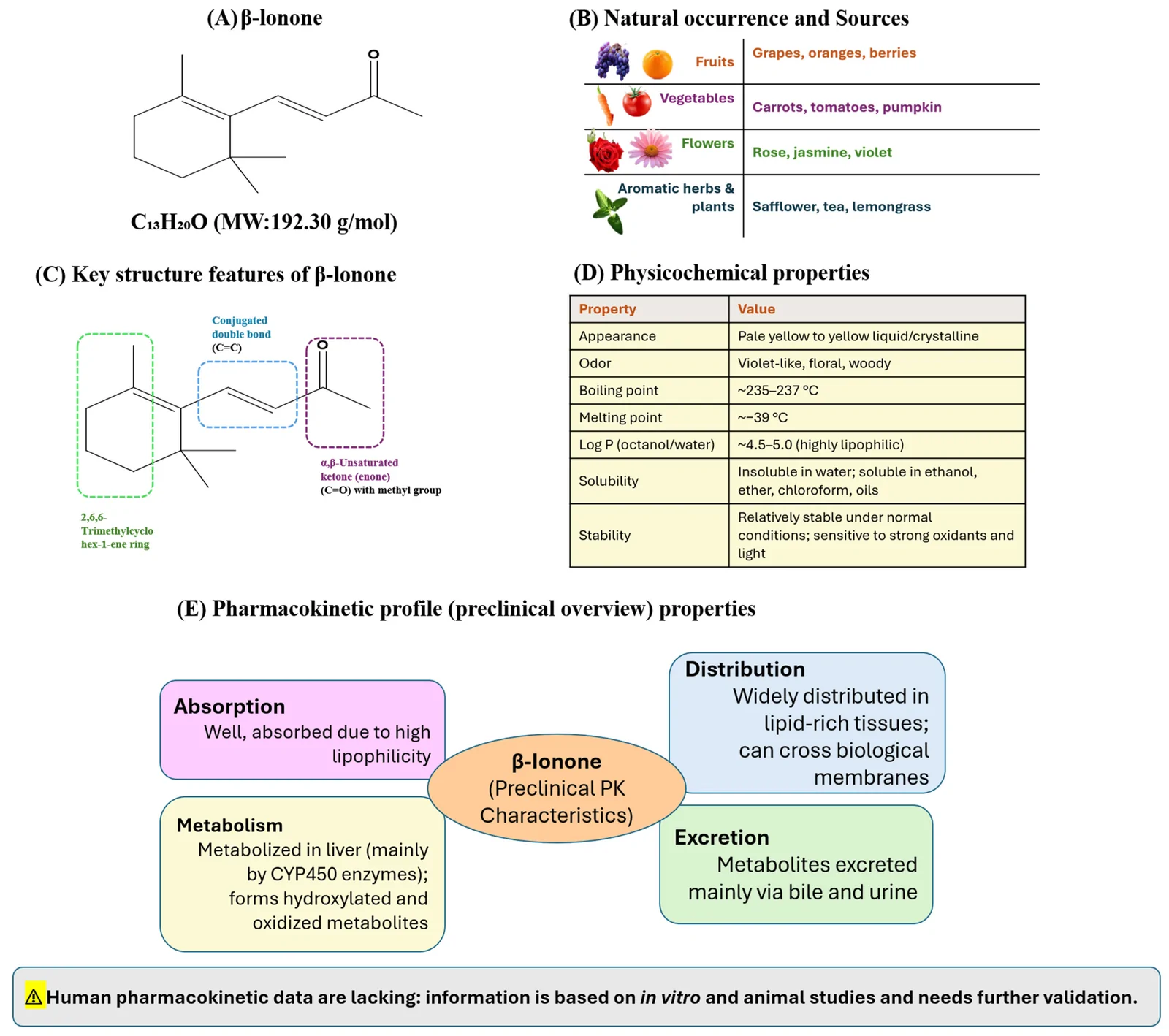
Chemistry, Structure–Activity Relationships (SAR) and Pharmacokinetics of β-ionone.

β-Ionone is an endogenous C13-norisoprenoid apocarotenoid that is produced by the oxidative or enzymatic cleavage of carotenoids, mostly β-carotene. β-Ionone is extensively present in fruits, vegetables, blossoms, and aromatic plants and is largely responsible for floral and fruity odors. Besides its relevance to the food, beverage, and cosmetic sectors, β-ionone has attracted attention among scientists for its biological roles, including antioxidant, anti-inflammatory, cancer-preventative, and antimicrobial activities [15,16]. β-Ionone is synthesized by enzymes known as carotenoid cleavage dioxygenases (CCDs), which catalyze the oxidative cleavage of carotenoid substrates, producing biologically functional apocarotenoids [17,18]. The chemical structure of β-ionone involves a cyclohexene ring connected with an unsaturated chain featuring a conjugated carbonyl functional group. The α, β-unsaturated ketone is considered a crucial pharmacophore for many of this compound’s biological activities (Figure 1). Molecular stability, lipophilicity, and biological interaction are attributed to the conjugated double bonds within the molecule, while the ketone plays a role in forming connections with other biomolecules. Experiments with derivatives of β-ionones revealed the importance of even small structural changes in the cyclohexene ring, side chain length, or degree of unsaturation in affecting biological function, highlighting the structure–activity relationship (SAR) within this compound family. The lipophilicity of β-ionone also enables it to interact with lipids and intracellular signaling proteins, which is believed to contribute to its anticancer activity [19,20]. The pharmacokinetic behaviour of β-ionone can be largely explained by its relatively small size and lipophilic nature. Being a volatile apocarotenoid, β-ionone has high membrane permeability and is likely to be absorbed quickly. This compound is characterized by good partitioning and should therefore be distributed effectively into lipid-enriched cellular structures, such as mitochondria, the key players in the induction of apoptosis. A proper balance between water and lipid solubility is one of the key predictors of bioavailability, cellular entry, and distribution [21]. In addition, structural features of β-ionone correlate well with criteria of ‘drug-likeness’, including Lipinski’s rule of five [22]. β-Ionone will likely undergo both phase I and phase II metabolism, including oxidation, reduction, and conjugation, facilitating its excretion from the body. While data on the pharmacokinetics of this compound are scarce, current knowledge of its physicochemical properties suggests it is suitable for adequate biological access and intracellular activity. Overall, the specific chemical structure, established structure–activity relationships, and favourable pharmacokinetic properties of β-ionone provide a solid foundation for exploring its potential as a functional compound with diverse biological activities.

It is important to distinguish between parent β-ionone and chemically modified β-ionone derivatives when interpreting the anticancer literature. Several β-ionone analogues, such as β-ionone-based esters, endoperoxides, and others, have been reported to exhibit significant cytotoxic or proapoptotic effects, but such molecules are not structurally identical to β-ionone and should not be interpreted as proof of β-ionone activity. For instance, β-ionone endoperoxides have been reported to exhibit significant cytotoxicity and ROS-mediated mitochondrial apoptosis in cancer-cell models, but these activities were observed with modified endoperoxide structures, not necessarily with β-ionone. Therefore, the present review divides the direct evidence for β-ionone from that for derivatives and structurally related compounds.

### Structure–Activity Relationship of β-Ionone and Its Derivatives

The anticancer property of β-ionone is linked with its unique C13-norisoprenoid architecture that includes a substituted cyclohexene ring and an unsaturated side-chain with a carbonyl group. These structural characteristics confer physicochemical properties and also serve as a scaffold that can be chemically modified to alter its biological properties [7,23]. Initial research showed that structural modification of the β-ionone framework can modulate its cancer-related activity, as β-ionone-derived alcohols exhibited biological activity in cancer chemoprevention models [24].

Sharma et al. (2014) also showed that they could synthesize β-ionone-derived endoperoxides, which exhibit improved cytotoxic and apoptosis-inducing effects in a range of cancer-cell models. The endoperoxide functionality was found to increase reactive oxygen species generation and mitochondrial apoptotic responses, demonstrating the significant effect of structural modification on the parent scaffold’s biological properties. More recently, a β-ionone derivative was shown to be DNA-binding, cytotoxic, and apoptosis-inducing in human K562 myeloid leukemia cells, in addition to inducing G0/G1 cell cycle arrest [12].

Taken together, these studies justify a qualitative SAR suggesting that substituting the β-ionone framework with an alcohol, ester, or endoperoxide group could affect cytotoxicity and the mechanism of action [12,24,25]. It is important to note that the increased activity observed with these derivatives is not directly attributable to the parent β-ionone, as the structural changes create chemically distinct derivatives. A systematic quantitative SAR is not yet available, however. Thus, additional analogue synthesis and biological evaluation are needed to confirm the key structure responsible for the anticancer activity of β-ionone [7,23].

## 4. Preclinical Evidence of Β-Ionone’s Anti-Cancer Effects

Preliminary studies demonstrate that β-ionone inhibits tumour cell growth and promotes cell apoptosis across various cancer types. Co-treatment with β-ionone inhibits SGC 7901 proliferation and promotes apoptotic cell morphology, suggesting dose-dependent activation of intrinsic apoptosis in human gastric adenocarcinoma SGC 7901 cells [26]. However, dietary β-ionone decreases PCNA and Bcl-2 expression and increases Bax expression in a rat model, indicating in vivo anticancer activity [27]. In prostate carcinoma cells, β-ionone functions as a proapoptotic and cell cycle-arresting agent by downregulating cyclin D1 and CDK4, thereby modulating tumor cell growth in vitro [28]. Additionally, β-ionone-induced apoptosis in human osteosarcoma U2OS cells was associated with a p53-dependent pathway and activation of intrinsic apoptotic signaling [29]. Together, these findings provide strong evidence for the potential of β-ionone as a natural anticancer agent with broad applicability and multiple modes of action (Table 1).

### 4.1. P53-Dependent Apoptosis

**Figure 2:**
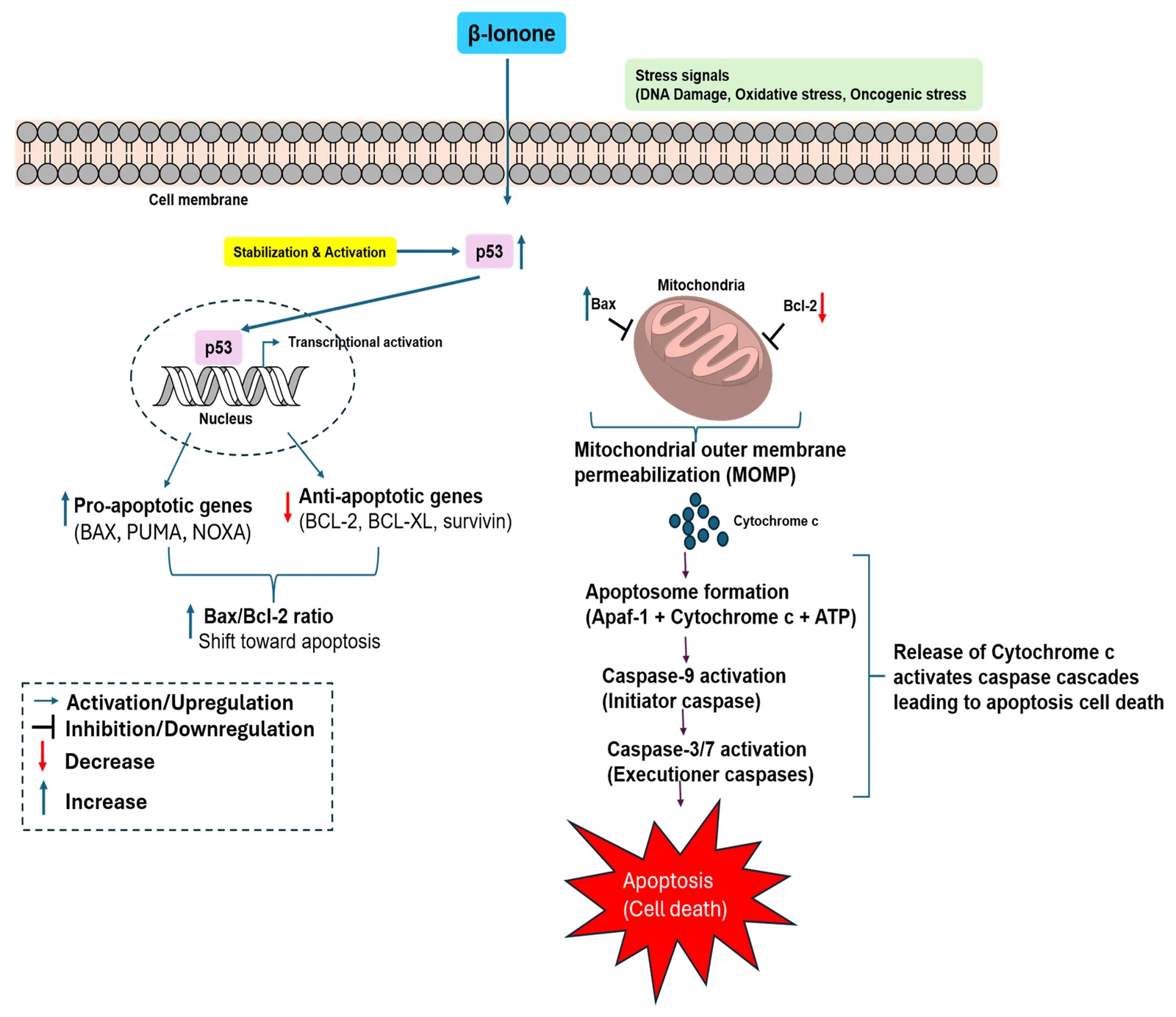
Proposed mechanism of β-ionone-induced p53-dependent mitochondrial apoptosis in cancer cells.

β-Ionone has been reported to induce apoptosis in several cancer cell models; however, the involvement of p53 appears to be tumor-model-dependent. In human U2OS osteosarcoma cells, β-ionone-induced apoptosis was specifically associated with a p53-dependent signaling pathway and mitochondrial apoptotic signaling [29]. In this model, alterations in Bax/Bcl-2 signaling and mitochondrial dysfunction were associated with apoptotic cell death. p53 activation results in enhanced transcription of pro-apoptotic cell death factors, such as BAX, and in inhibition of anti-apoptotic factors, such as BCL-2. This process increases the permeability of the mitochondrial outer membrane, leading to the release of cytochrome c. When Apaf-1 and procaspase-9 bind in the cytosol, they form the Apoptosome, which activates caspase-9 and downstream executioner caspases such as caspase-3. This cascade ultimately causes DNA fragmentation and triggers programmed cell death [30,31]. In human gastric adenocarcinoma cancer SGC-7901 cells, β-ionone induced apoptosis through a PI3K-AKT pathway and apoptotic signaling activation [32]. In contrast, β-ionone-induced apoptosis has also been reported in SGC-7901 gastric carcinoma cells; however, direct involvement of p53 was not established in that model [33]. Overall, these data indicate that β-ionone might interact with p53-regulated apoptotic pathways and trigger mitochondrial cell death, although additional research is required to measure the dynamics of p53 activation and its transcriptomic output across various cancer types. β-Ionone-induced activation of p53 promotes mitochondrial apoptosis through regulation of Bax, Bcl-2, cytochrome c release, and caspase activation, as illustrated in Figure 2 and Table 1.

### 4.2. Modulating the Bcl2-Bax Pathway

An optimal dose of β-ionone modulates cancerous cells, preventing or reducing tumour growth by stimulating apoptosis (programmed cell death). In general, modulation of the Bcl-2/Bax pathway is a more efficient way to regulate cell survival and death rates. It was reported that β-ionone downregulates Bcl-2 gene expression (an anti-apoptotic protein), whereas it upregulates Bax gene expression (a pro-apoptotic protein) [27]. However, this change in the Bcl-2/Bax gene expression ratio disrupts mitochondrial membrane integrity. A disturbance in the outer mitochondrial membrane leads to mitochondrial outer membrane permeabilization (MOMP). MOMP releases cytochrome c, which binds to the Apaf-1 (apoptotic protease-activating factor-1) protein and procaspase-9, forming a complex known as the apoptosome. This apoptosome further activates caspase-9, which in turn stimulates downstream caspases, such as caspase-3 and caspase-7, leading to apoptosis. Moreover, β-ionone could suppress the initiation and progression of cancer by arresting the cell cycle and preventing the metastatic spread of unwanted cells [12]. It could be reduced by altering cell-signalling pathways involved in cell migration and survival. Simultaneously, the sensitivity of cancerous cells to chemotherapy could be enhanced using β-ionone to overcome drug resistance. Cancer research findings demonstrated that β-ionone is an effective natural compound for controlling cancer cell proliferation and tumour growth through intrinsic apoptotic mechanisms [27]. Thus, β-ionone has potential for cancer therapy, but further empirical studies are required to determine the appropriate dosage and timing of this compound for therapeutic and diagnostic success in humans.

### 4.3. Caspase Cascade Activation

The activation of the caspase cascade is tightly coupled to β-ionone-induced apoptosis and serves as the key execution pathway in cancer cell apoptosis [26]. β-Ionone also suppressed proliferation and induced apoptotic morphology in human gastric adenocarcinoma SGC-7901 cells, indicating activation of downstream apoptotic signaling mechanisms that involve caspase-dependent cell death [26].

Further studies showed that β-ionone could induce apoptosis in the gastrocarcinoma cell line SGC-7901 in a dose-dependent manner by inhibiting the PI3K/Akt signalling pathway and activating caspase-3 [32]. Caspase-3 is a key executioner caspase that cleaves several structural and regulatory substrates, leading to the characteristic morphological and biochemical changes in apoptosis, such as chromatin condensation and DNA fragmentation [32]. The study demonstrated that β-ionone sensitised prostate cancer cells that express the death receptor, thereby potentially boosting the activity of initiator caspases in the extrinsic apoptotic pathway, while also inhibiting the cell cycle and causing death of prostate cancer cells [28]. β-Ionone also enhanced TRAIL-induced apoptosis, suggesting its potential to augment death receptor-mediated apoptotic signalling in hormone-related cancers [34]. In osteosarcoma U2OS cells, however, β-ionone induced apoptosis via a p53-dependent mechanism, possibly through increased ROS generation and subsequent caspase activation [29]. β-Ionone-oriented proapoptotic derivatives also induced additional ROS generation and subsequent caspase activation, leading to mitochondrial injury and apoptotic cell death [10]. All these results point to β-ionone’s ability to trigger both intrinsic and extrinsic apoptotic pathways, culminating in common final events such as caspase-3 activation and, consequently, apoptosis [7].

### 4.4. Targeting the PI3K-Akt-mTOR Pathway

The phosphatidylinositol 3-kinase (PI3K)/Akt/mTOR signalling pathway is often aberrantly regulated in cancer, significantly impacting cancer cell proliferation, metabolism, angiogenesis, and resistance to apoptosis. Abnormal activation of PI3K, Akt, or mTOR enhances cellular survival by activating anti-apoptotic proteins, suppressing pro-apoptotic mediators, and promoting protein synthesis and cell-cycle progression. As a result, inhibition of this pathway has become a significant therapeutic approach to restore apoptotic sensitivity in cancer cells [35]. β-ionone, a naturally derived apocarotenoid, has exhibited remarkable anti-cancer efficacy via regulating the PI3K/Akt signalling pathway. One of the initial mechanistic experiments indicated that β-ionone triggered apoptosis in SGC-7901 gastric cancer cells by suppressing PI3K expression and phosphorylated Akt. This suppression was correlated with enhanced caspase-3 activation and reduced expression of the anti-apoptotic protein Bcl-2, suggesting that inhibition of the PI3K/Akt signalling pathway directly facilitates β-ionone-induced apoptotic cell death. Additionally, β-ionone administration markedly increased the proportion of apoptotic cells in a dose-dependent manner, indicating that blocking survival signalling is a primary mechanism underlying its anti-cancer efficacy [32]. Recent advancements in cancer biology have shown that the PI3K/Akt/mTOR pathway is a central regulator linking growth factor signalling to apoptosis and autophagy [36]. This pathway is inhibited; mTOR activity and cellular protein synthesis are reduced; cells stop growing; and innate apoptotic mechanisms are activated. Inhibiting the PI3K/Akt/mTOR pathway has emerged from recent studies as a pro-apoptotic strategy. Experimental medications that suppress Akt phosphorylation and mTOR activation have been reported to synergistically trigger apoptosis and autophagy in non-small cell lung cancer and drug-resistant cancers. These findings are particularly pertinent to β-ionone, as its inhibition of PI3K/Akt signaling suggests extensive downstream effects on mTOR-dependent survival pathways. However, direct evidence for β-ionone-mediated mTOR inhibition remains limited and warrants further research [37,38,39].

### 4.5. Mapk/Erk Modulation

**Figure 3:**
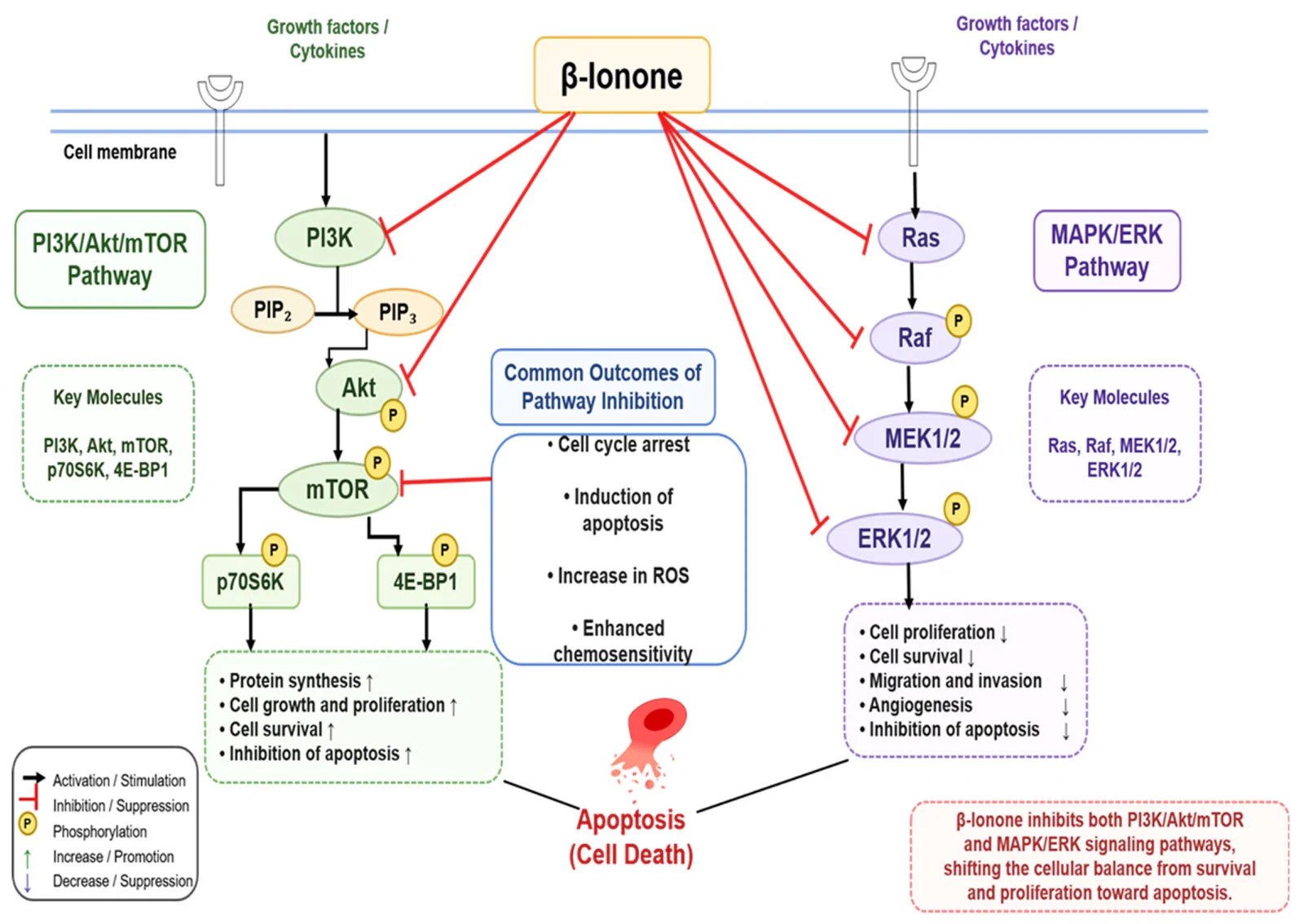
β-Ionone-mediated modulation of PI3K/Akt/mTOR and MAPK/ERK signaling pathways in cancer cells.

β-Ionone inhibits both PI3K/Akt/mTOR and MAPK/ERK signalling pathways, as summarised in Figure 3. β-Ionone modulates the MAPK/ERK signalling axis in cancer cells, and this pathway is closely linked with tumour cell proliferation, cell-cycle progression, apoptosis, invasion, and therapeutic resistance [40]. In gastric carcinoma cells, β-ionone was shown to arrest the cell cycle through a MAPK-associated mechanism, suggesting that MAPK modulation contributes to its antiproliferative activity [40]. This MAPK-mediated cell-cycle arrest was associated with the transcriptional downregulation of key cell-cycle proteins, thereby limiting uncontrolled proliferation of gastric cancer cells [40]. In colorectal cancer, β-ionone acts as an OR51E2 ligand, increasing intracellular calcium levels, thereby suppressing MEK/ERK phosphorylation and promoting apoptosis [41]. The inhibitory effect of β-ionone on colorectal cancer cell proliferation was abolished after OR51E2 knockdown, indicating that OR51E2 is an important upstream regulator of β-ionone-mediated MEK/ERK suppression [41]. In xenograft models, β-ionone administration significantly reduced colorectal tumor growth, further supporting the therapeutic relevance of OR51E2-linked MEK/ERK inhibition [41]. However, in prostate cancer cells, β-ionone can activate ERK1/2 via OR51E2 via a Golgi-localised Gβγ-PI3Kγ-ARF1 signalling pathway, demonstrating that its MAPK/ERK effects are context-dependent [42]. This prostate cancer study demonstrated that β-ionone-induced ERK1/2 activation was blocked by inhibition or knockout of Gβγ, PI3Kγ, or ARF1, confirming pathway-specific regulation [42]. Therefore, β-ionone may either suppress or activate MAPK/ERK signalling, depending on tumour type, receptor expression, and intracellular signalling context [40,41,42]. Collectively, these findings indicate that MAPK/ERK modulation is an important, cancer-specific mechanism that contributes to β-ionone-mediated growth inhibition, apoptosis, and cell-cycle regulation [40,41].

### 4.6. ROS-Mediated Apoptosis

β-Ionone-mediated apoptosis is strongly associated with reactive oxygen species (ROS) generation, which can promote oxidative stress, mitochondrial dysfunction, and activation of apoptotic signaling in cancer cells [10]. Proapoptotic activity is associated with enhanced ROS generation, an important mechanism underlying the anti-cancer and proapoptotic activity of β-ionone-oriented agents [10]. Excessive intracellular ROS may damage the mitochondrial membrane, decrease mitochondrial membrane potential, and induce cytochrome c release, thereby linking intrinsic mitochondrial apoptosis to oxidative stress (OS) [43]. In the cytoplasm, cytochrome c participates in the formation of apoptosomes and triggers apoptosis by activating the caspase cascade, linking ROS accumulation to caspase activation [43]. Additionally, ROS have been identified as crucial regulators of apoptosis as they can modulate apoptotic pathways involving mitochondria, death receptors, and endoplasmic reticulum stress [44]. In cancer models treated with β-ionone, the elevation in ROS generation can work with Bax activation and Bcl-2 suppression to tip the intracellular balance towards increased permeability of the outer mitochondrial membrane [10]. Mitochondrial injury further increases the activation of executioner caspases, such as caspase-3, leading to the typical features of apoptosis, such as DNA fragmentation and chromatin condensation [43]. β-Ionone-derived endoperoxides have also been reported to induce apoptosis through the mitochondrial pathway, supporting the importance of oxidative and mitochondrial mechanisms in β-ionone-related compounds [25]. Because ROS levels can determine whether cancer cells undergo survival adaptation or apoptotic collapse, β-ionone-induced ROS production may represent a key mechanism for overcoming apoptosis resistance [44]. Collectively, available evidence suggests that ROS-mediated mitochondrial damage is an important contributor to β-ionone-induced apoptosis and may enhance its potential as a natural pro-apoptotic anti-cancer agent [7,10].

### 4.7. Fas-Fasl Pathway Activation

In several fruits and vegetables, a natural apocarotenoid, i.e., β-Ionone, has been shown to have anti-cancer properties [7]. The Fas-Fas Ligand (FasL) pathway is an important extrinsic apoptotic pathway implicated in the anticancer activity of β-ionone. CD95/APO-1 is a death Fas receptor that occurs on cancer cells, which associates with FasL to cause programmed cell death or apoptosis [45]. β-Ionone has been reported to enhance Fas receptor/FasL-associated apoptotic signaling, thereby promoting apoptosis in cancer cells [16]. The association between FasR and FasL promotes formation of the death-inducing signaling complex (DISC), involving the adaptor protein Fas-associated death domain (FADD), which activates the initiator caspase-8. This subsequently triggers a caspase cascade involving caspase-3 and caspase-7, ultimately contributing to DNA fragmentation and apoptotic cell death [46]. Moreover, β-ionone activates both the extrinsic (death receptor) and intrinsic (mitochondrial) apoptotic pathways. In this pathway, activated caspase-8 can cleave Bid, producing truncated Bid (tBid), which disrupts mitochondrial membrane permeability and releases cytochrome C. It stimulates the caspase cascade and amplifies apoptosis [47]. Activation of the FasR-FasL pathway by β-ionone may therefore contribute to the induction of extrinsic apoptosis and amplification of apoptotic signaling [48]. Available preclinical evidence suggests that β-ionone may modulate FasR-mediated signalling and caspase-dependent apoptotic pathways; however, further studies are required to establish the role of this pathway across different tumor models. Further stochastic and empirical studies are still required to elucidate its molecular characteristics and clinical applications. The death receptor pathway has been primarily investigated in hepatocellular carcinoma models, in which β-ionone enhanced TRAIL-induced apoptosis by increasing DR5 expression and suppressing NF-κB activity [34].

### 4.8. Targeting the Jak-Stat3 Signalling Pathway

The Janus kinase/signal transducer and activator of transcription 3 (JAK-STAT3) pathway represent a potential, but currently unvalidated, mechanism underlying β-ionone-mediated anticancer activity. Although STAT3 is an established regulator of cancer cell survival, proliferation, and resistance to apoptosis, direct experimental evidence for β-ionone-mediated JAK-STAT3 inhibition remains limited [49]. Constitutive STAT3 activation has been shown in various carcinomas, including breast, lung, gastric, colorectal, and haematological cancers, where it promotes tumour progression by transcriptionally activating genes linked to cell survival, such as Bcl2, MCL1, Bcl-XL, cyclin D1, and survivin [50]. Prolonged STAT3 activation further promotes resistance to chemotherapy and apoptosis, suggesting this pathway is a promising target for anti-cancer strategies. β-Ionone primarily induces apoptosis through p53-dependent mitochondrial pathways, characterized by upregulation of Bax, downregulation of Bcl2, stimulation of cytochrome c release, and activation of caspase-3. Furthermore, COX2 inhibition in breast cancer, Wnt/β-catenin inhibition that triggers ferroptosis in gastric cancer, and OR51E2 activation linked with MEK-ERK suppression in colorectal cancer are also significant. Recent evidence indicates that phytochemicals proactively target JAK/STAT3 signaling [7]. Several naturally occurring terpenoids and carotenoid-derived compounds chemically related to β-ionone have been shown to suppress STAT3 phosphorylation, thereby decreasing the production of anti-apoptotic proteins and increasing apoptotic cell death in cancer [51,52]. STAT3-mediated transcription is intricately linked to the maintenance of an anti-apoptotic cellular environment. Hence, suppression of JAK-STAT3 signaling can restore apoptotic sensitivity by reducing the expression of survival proteins and facilitating caspase activation. Given the documented role of β-ionone in inducing apoptosis by regulating several survival pathways, additional studies are required to determine whether direct targeting of JAK-STAT3 enhances its anti-cancer effects. Understanding this mechanism may provide further insights into the multifaceted therapeutic potential of β-ionone in cancer therapy [53]. Therefore, although JAK-STAT3 represents a biologically plausible target based on evidence from related phytochemicals and cancer signaling studies, direct confirmation in β-ionone-treated cancer models is required before considering this pathway as an established mechanism.

## 5. β-Ionone and Non-Apoptotic Cell Death Mechanisms

### 5.1. Autophagy as a Proposed Mechanism of β-Ionone-Induced Cell Death

The role of autophagy in the anticancer activity of β-ionone remains poorly understood. The evidence available is largely mechanistic in nature; it is based on association with oxidative stress, mitochondrial dysfunction, Bcl-2 regulation, and PI3K/Akt/mTOR signaling, rather than direct experiments with β-ionone-specific markers of autophagy or pharmacological and genetic inhibition approaches. So, autophagy should be considered a potential, but not proven, mode of cell death induced by β-ionone at this point. Biologically, there is a possibility for interactions between apoptosis and autophagy given that both cellular processes are regulated by common elements such as Beclin-1, Bcl-2 family proteins, and the PI3K/Akt/mTOR signaling pathway. However, the role of autophagy as a cytoprotective response or as a direct cause of cell death after β-ionone exposure has yet to be determined. To conclude, further research with LC3-II/p62 analysis, autophagic flux assays, specific autophagy inhibitors, and genetic manipulation will be needed to fully elucidate the role of autophagy in cancer cells treated with β-ionone. Therefore, autophagy should currently be considered a proposed mechanism requiring validation in specific cancer models [54]. β-Ionone exerts potent apoptotic effects through several pathways, including the generation of reactive oxygen species (ROS), loss of the mitochondrial membrane potential (MMP), and p53-dependent pathways. Moreover, β-ionone derivatives with greater ROS-generating potential exhibit enhanced cytotoxicity, leading to redox imbalance and oxidative stress, as evidenced by lipid peroxidation, reduced MMP levels, Bax and caspase-3 activation, and cell apoptosis. Increased mitochondrial ROS production activates the apoptosis and autophagy pathways, which may lead to cell death [29]. The interaction between autophagy and apoptosis is regulated by several common molecular regulators, including Beclin-1, Bcl-2 family proteins, and the PI3K/Akt/mTOR signalling pathways. Given the complex regulation of autophagic activity by signals emanating from the PI3K/Akt/mTOR pathway and the modulation of the gene expression of pro- and anti-apoptotic proteins, such as Bcl-2, by β-ionone treatment, this compound could indirectly affect autophagic activity through these mechanisms. The downregulation of Bcl-2 can relieve inhibition of Beclin-1, thereby promoting the initiation of autophagy and enhancing the interaction between autophagy and apoptosis [55]. There is still limited direct evidence that the apoptosis effects of β-ionone are correlated with changes in autophagy. The available evidence suggests that autophagy may contribute to β-ionone-mediated anticancer effects; however, whether autophagy acts as a cytoprotective response or a cell death-promoting mechanism remains unresolved. Future studies using LC3-II/p62 analysis, autophagy inhibitors, and genetic approaches are required to establish the functional role of autophagy in β-ionone-treated cancer cells [56].

### 5.2. Ferroptosis

**Figure 4:**
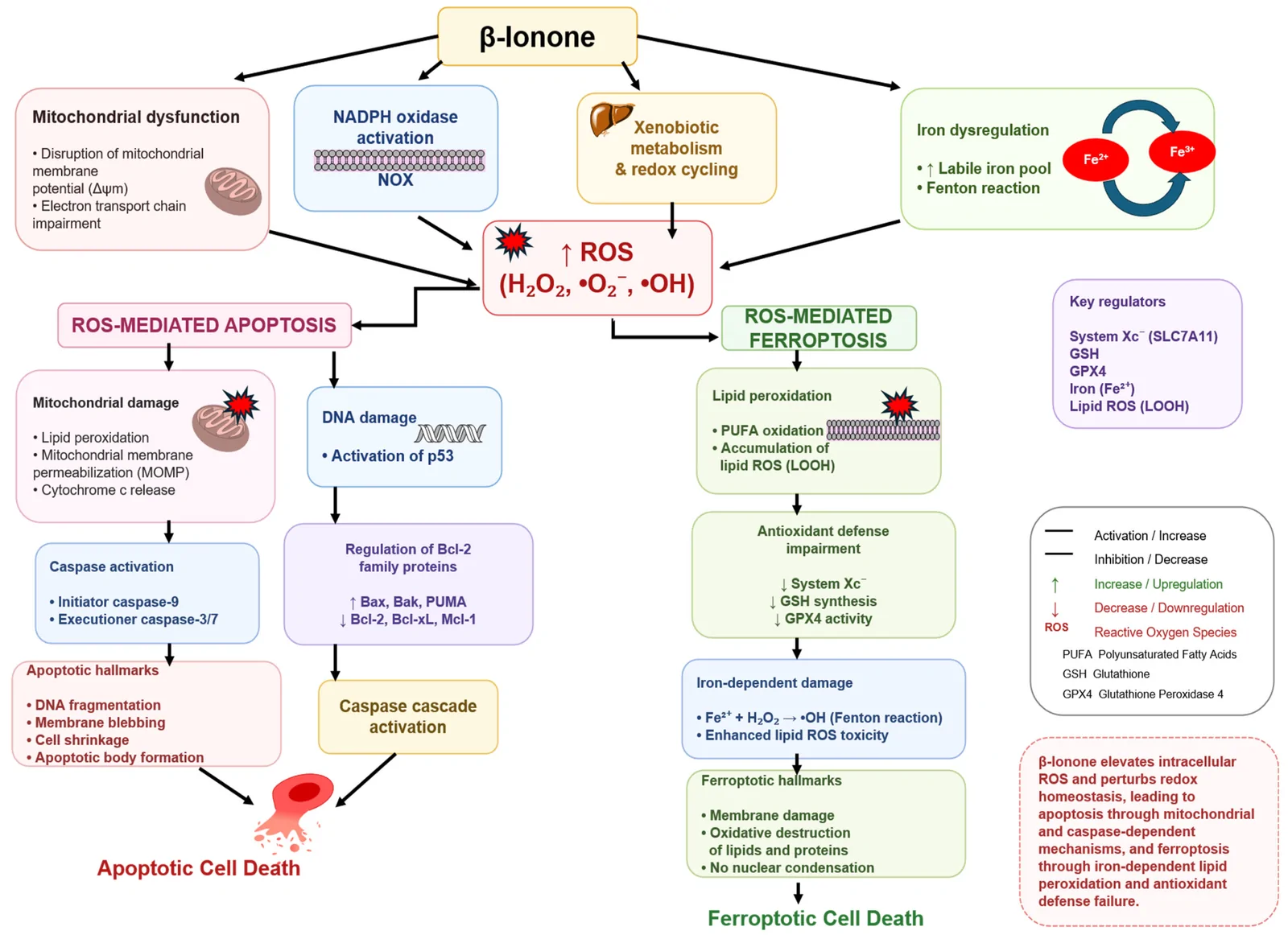
ROS-mediated apoptosis and ferroptosis induced by β-ionone in cancer cells.

Ferroptosis is an iron-mediated mode of cell death that involves excess ROS production and lipid peroxidation and does not require classical apoptosis or necrosis [57]. Recent evidence from gastric cancer models demonstrates that β-ionone induces ferroptosis through inhibition of Wnt/β-catenin signaling and reduction in ferroptosis-regulating proteins such as GPX4. In human gastric cancer cells and xenograft models, β-ionone increased oxidative stress and lipid peroxidation, contributing to tumor growth suppression [41]. By stimulating ferroptosis, β-ionone suppresses Wnt/β-catenin signalling and reduces glutathione peroxidase 4 (GPX4) expression, an important regulator of lipid hydroperoxide detoxification [58]. The accumulation of iron is also linked to β-ionone-induced ferroptosis, as high intracellular Fe^2+^ promotes Fenton reactions, intensifying oxidative stress and lipid peroxidation in cancer cells, alongside other apoptotic pathways, particularly in those that lack sensitivity to apoptosis [59]. β-Ionone derivatives with superior pro-oxidant activity further contribute to redox imbalance and trigger ferroptotic cell death, in addition to activating apoptosis pathways, especially in cancer types resistant to apoptosis [10]. This ROS-dependent ferroptotic pathway is another complementary mechanism by which β-ionone inhibits tumor growth and is resistant to drug resistance, suggesting it could be a viable synergistic therapeutic approach with conventional chemotherapy drugs [41]. Recent experimental evidence strongly supports ferroptosis as a mechanism of action of β-ionone in gastric cancer, as Fe^2+^ accumulation, total and lipid ROS levels, and malondialdehyde levels were augmented in MKN45 and AGS cells, and mitochondrial membrane potential and SLC7A11 and GPX4 expression were decreased [60]. In xenograft models, β-ionone also inhibited tumor growth and decreased proteins associated with ferroptosis. Mechanistically, this was attributed to GSK-3β inhibitors partially abolishing β-ionone’s effects on ferroptosis and cell-cycle regulation [60]. Therefore, ferroptosis may be a cancer model-dependent mechanism of action for β-ionone in experimental studies, but not a general pathway. The proposed ROS-mediated apoptotic and ferroptotic mechanisms of β-ionone are summarized in Figure 4.

### 5.3. Cell Cycle Arrest

β-Ionone’s ability to inhibit the growth of cancer cells partially involves cell-cycle arrest, which restrains uncontrolled proliferation before apoptotic cell death [28]. In human prostate tumor cells, β-ionone induced both growth arrest and apoptosis, suggesting that growth inhibition and programmed cell death are coordinated anti-cancer activities of β-ionone [28]. β-Ionone treatment is reported to target growth-inhibitory proteins, such as cyclins and cyclin-dependent kinases, necessary for progression through the G1/S and G2/M checkpoints [28]. In gastric carcinoma cells, it was shown that β-ionone arrested the cell cycle by suppressing the mitogenic signaling pathway associated with MAPK. In gastric carcinoma cells, suppression of cell-cycle progression has been shown to be associated with altered mitogenic signalling via the MAPK pathway. In this study, gastric cancer was examined, and it was suggested that β-ionone induced cell-cycle arrest by downregulating the transcription of cell-cycle proteins, thereby decreasing proliferative capacity. In gastric cancer cells, β-ionone can inhibit cell cycle progression and promote apoptosis by modulating MAPK activity, thereby inhibiting tumor growth and increasing cell sensitivity to apoptosis induction [40]. Earlier work in SGC-7901 gastric adenocarcinoma cells also showed that β-ionone inhibits cancer cell proliferation and induces apoptosis, supporting its role as a growth-suppressive natural compound [26]. β-Ionone-induced cell-cycle arrest may also cooperate with mitochondrial apoptotic mechanisms, including modulation of the Bax/Bcl-2 ratio, cytochrome c release, and caspase activation [32]. When combined with other anti-cancer agents, β-ionone may enhance therapeutic response by suppressing proliferation and promoting apoptosis, although more direct cell-cycle checkpoint studies are still needed [26,32]. Overall, available evidence suggests that β-ionone-mediated cell-cycle arrest is an important antiproliferative mechanism that complements its pro-apoptotic effects in cancer cells. To provide a cancer-specific overview of available evidence, Table 1 summarizes original experimental studies evaluating β-ionone-mediated anticancer effects, including cancer models, treatment conditions, molecular targets, and biological outcomes.

**Table 1:** Cancer-specific experimental evidence of β-ionone-mediated anticancer activity.

Cancer Type	Experimental Model	β-Ionone Treatment/Dose	Molecular Targets and Pathways	Major Outcomes	Study Type	Reference
Gastric adenocarcinoma	Human gastric adenocarcinoma SGC-7901 cells	25, 50, 100, and 200 μM; 24 and 48 h; IC_50_ ≈ 89 μM	Apoptosis; apoptotic morphology; TUNEL-positive cells	Concentration- and time-dependent inhibition of cell growth and induction of apoptosis	In vitro	[26]
Gastric carcinoma	SGC-7901 gastric carcinoma cells	25, 50, 100, and 200 μM; 24 h	PI3K/AKT pathway; cleaved caspase-3; Bcl-2	Dose-dependent apoptosis, suppression of PI3K/AKT signaling, increased caspase-3 activation and decreased Bcl-2	In vitro	[32]
Mammary carcinoma	DMBA-induced mammary carcinogenesis in Sprague-Dawley rats	9, 18, and 36 mmol/kg diet, beginning 2 weeks before DMBA and continuing for 24 weeks	PCNA; cyclin D1; Bax/Bcl-2; apoptotic signaling	Dose-dependent suppression of mammary carcinogenesis, reduced proliferation, increased apoptosis, delayed tumor appearance and reduced tumor multiplicity	In vivo	[27]
Prostate cancer	Human DU145, LNCaP, and PC-3 prostate cancer cells	IC_50_: 210 μM (DU145), 130 μM (LNCaP), 130 μM (PC-3); β-ionone 150 μM was also used in combination experiments	Cyclin D1; CDK4; HMG-CoA reductase/mevalonate pathway; G1 cell-cycle regulation	Inhibited proliferation, induced G1 cell-cycle arrest and apoptosis, and reduced cyclin D1/CDK4 and HMG-CoA reductase expression	In vitro	[28]
Osteosarcoma	Human U2OS osteosarcoma cells	50, 100, 150, 200, 250, 300, 350, and 400 μM; 48 h; IC_50_ ≈ 175 μM; 200 μM used as an apoptosis-induction condition	p53-dependent mitochondrial pathway; Bax/Bcl-2; cytochrome c; caspase-3	Concentration- and time-dependent inhibition of proliferation, G1/S arrest and apoptosis	In vitro	[29]
Hepatocellular carcinoma	Hep3B and HepG2 hepatocellular carcinoma cells	β-Ionone tested at increasing concentrations up to 400 μM; 200 μM used for mechanistic studies; combined with 100 ng/mL TRAIL, generally for 24 h	Sp1/DR5; NF-κB; caspase-8/-9/-3; mitochondrial apoptotic pathway	Sensitized HCC cells to TRAIL-induced apoptosis, increased DR5 and suppressed TRAIL-associated NF-κB signaling	In vitro	[34]
Leukemia	Human chronic myelogenous leukemia K562 cells	25, 50, 100, 200, 300, and 400 μM; 24, 48, 72, and 96 h; apoptosis assessed mainly at 25–200 μM for 24 h	Apoptosis-associated pathways; Annexin V/PI; nuclear condensation/fragmentation	Dose- and time-dependent inhibition of proliferation and increased apoptotic cell death	In vitro	[11]
Myeloid leukemia—β-ionone derivative	Human K562 myeloid leukemia cells	β-Ionone-derived ester 4-TM.P: 10–100 μM; 24–72 h; IC_50_ = 65 ± 5 μM (24 h), 50 ± 4 μM (48 h), 25 ± 5 μM (72 h)	DNA binding; G0/G1 arrest; caspase-3; apoptosis	Inhibited K562 cell growth and induced apoptosis and G0/G1 arrest	In vitro	[12]
Gastric cancer	Human MKN45 and AGS gastric cancer cells; gastric cancer xenograft model (BALB/c nude mice)	50–1000 μM/L in cells and 50 mg/kg body weight (b.w.) was used in vivo	Wnt/β-catenin; GSK-3β; SLC7A11; GPX4; ferroptosis; Cyclin D1/CDK4	Reduced cell viability and tumor growth, increased ferroptosis-associated oxidative stress and Fe^2+^/lipid ROS, and decreased GPX4/SLC7A11 and Wnt/β-catenin signaling	In vitro and in vivo	[60]
Colorectal cancer	OR51E2-expressing colorectal cancer models, including SW620 cells and nude-mouse xenografts	200 μM associated with ~50% inhibition of migration; 400 μM reduced colony formation by ~40%; in vivo 5 and 10 mg/kg body weight	OR51E2 activation; intracellular Ca^2+^; MEK/ERK signaling	Reduced proliferation, colony formation and migration; induced apoptosis; suppressed xenograft tumor growth	In vitro and in vivo	[41]

## 6. Synergistic and Nanodelivery Aspects

**Figure 5:**
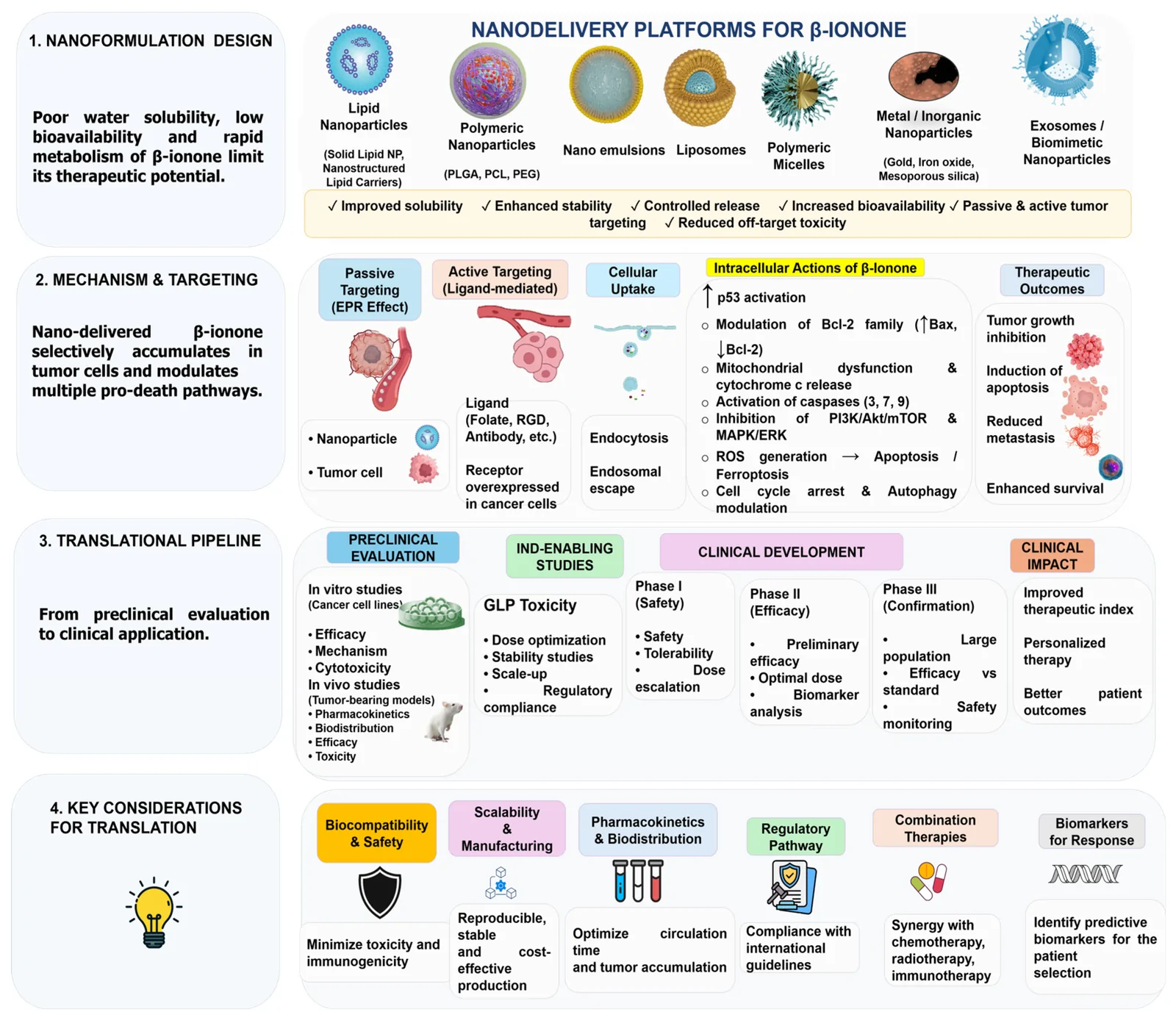
The nanodelivery and the schematic translation of the compound β-ionone in cancer treatment.

The use of natural bioactive compounds in medicine is often limited by factors such as limited aqueous solubility, rapid metabolism, low bioavailability, and insufficient drug accumulation at cancer sites [61]. While β-ionone is expected to exhibit excellent anticancer activity due to its ability to modulate apoptotic and survival mechanisms, its application may be limited by the compound’s properties and behaviour in the human body [7]. Modern nanotechnologies can help address some of these problems and promote the development of more efficient treatment options. To achieve better drug stability, controlled release, targeted delivery, and intracellular transport, various nanocarrier delivery systems, such as liposomes, polymeric, micellar, dendritic, and lipid-based nanoparticles, have been investigated [62,63]. Cancer research can benefit from nanomedicines, which can be specifically delivered to tumours via the EPR effect and more effectively target cancer cells through active targeting. Furthermore, the functionalized nanocarriers can be used to reduce drug toxicity and enhance drug pharmacokinetics. Additionally, stimuli-responsive nanocarriers are known to be promising nanoplatforms that release cargo under tumor-specific conditions, such as low pH, oxidative conditions, enzymatic degradation, and temperature changes [64]. Natural compound-based combination therapies have also attracted significant interest as a means of enhancing the efficacy of conventional treatments for various cancers. The simultaneous modulation of multiple signaling pathways and molecular targets by natural products makes them useful tools in anti-cancer therapy. In particular, β-ionone and some of its derivatives exhibit strong antiproliferative and pro-apoptotic properties and may be used in combination therapy schemes as novel anti-cancer drugs [7]. Moreover, the use of numerous bioactive plant compounds in contemporary medicine underscores their high potential for developing new anticancer drugs [6]. Though there is little evidence for β-ionone-containing nanocarriers, the positive physicochemical and biological properties of this molecule render it a candidate for nanoformulations. Thus, further studies are needed to evaluate the potential benefits of β-ionone in a targeted form. The design of β-ionone nanocarriers and their translational pipeline are summarized in Figure 5.

## 7. Therapeutic Implications of β-Ionone in Cancer Management

The preclinical results indicate several therapeutic applications for β-ionone in cancer management [65]. Given its ability to inhibit multiple cancer-related pathways, β-ionone could be a multi-target anticancer compound [60,65]. In addition to inhibiting cancer cell proliferation, it could enhance their apoptotic sensitivity and reverse certain resistance mechanisms [60]. Mechanistically, it can inhibit mitochondrial apoptosis, modulate the PI3K/Akt survival signalling cascades, respond to oxidative stress, and retard the cell cycle, supporting its potential for therapeutic exploration [7]. β-Ionone may be useful as a combination therapy agent rather than as a single anti-cancer therapeutic [9]. Other studies have shown that β-ionone can augment the effect of TRAIL in hepatocellular carcinoma models, suggesting that it can increase susceptibility to existing anti-cancer methods [34]. Hence, combination treatment with chemotherapy, targeted drugs, or other pro-apoptotic agents using β-ionone could be an interesting area to explore in the future [9]. Nanotechnology strategies may improve therapeutic effectiveness and reduce systemic exposure. Although these potential applications are very favourable, β-ionone remains in its preclinical stage, and many hurdles that require research, such as pharmacokinetic characterisation and optimisation, toxicity assessment, and clinical confirmation, must be overcome before it becomes a therapeutic option.

## 8. Limitations and Challenges Associated with β-Ionone-Based Cancer Therapy

Despite its potential as a potent, preclinical anticancer agent, β-ionone has several drawbacks. Most data come from in vitro cancer cell studies, with limited in vivo data and no clinical studies to confirm these findings. There is no experimental data to indicate relationships between effective concentrations and exposures in humans, requiring pharmacokinetic–pharmacodynamic studies. There are areas of knowledge that are not yet fully understood, such as the absorption, distribution, metabolism, and excretion of β-ionone. Its physicochemical properties are well-suited for cellular interactions, but the lack of solubility and bioavailability and limited systemic reach may affect its development [7]. There is limited safety information, particularly regarding chronic effects, organ-specific effects, and interactions. Furthermore, the suggested mechanisms, including the p53-mediated apoptotic pathway, PI3K/Akt modulation, MAPK/ERK pathway regulation, and ROS-mediated pathways, need to be tested specifically to confirm their roles in lung cancer [10]. The lack of clinical trials is the reason for the translational gap. Pharmacokinetic and toxicity studies, as well as biomarker and formulation studies, are required for future studies to examine the potential of β-ionone in oncology.

## 9. Safety Aspects, Pharmacokinetic Gaps, Translational Barriers and Future Perspectives

Although β-ionone shows great promise as an anti-cancer agent, it is not used in clinical practice due to insufficient safety and pharmacokinetic data [6]. In vitro and in vivo studies have shown low acute toxicity; however, chronic exposure, organ-specific toxicity, and off-target effects remain to be fully explored [10]. A major limitation in translating β-ionone from experimental studies to clinical application is the lack of comprehensive pharmacokinetic characterisation. Although in vitro studies demonstrate anticancer activity at experimental concentrations, it remains unclear whether therapeutically relevant plasma or tumor tissue concentrations can be achieved following systemic administration. Factors including poor aqueous solubility, rapid metabolism, protein binding, and limited tumour accumulation may influence its effective bioavailability. Information about the pharmacokinetics of β-ionone is limited, with only a few studies available. It is characterized as highly lipophilic and membrane-permeable, resulting in rapid absorption and cellular uptake; however, its metabolism and clearance rates in humans remain poorly characterized and are yet to be determined [41]. The formulation challenges of low aqueous solubility and short half-life limit systemic delivery and tumor targeting; this highlights the need for nanoformulations and/or carrier-based strategies [66]. Among the translational barriers are the following: the treatment has different efficacy across cancers, there is limited knowledge of molecular targets, and there is a lack of dose-titration studies [67]. Safety evaluation of β-ionone remains incomplete. Available experimental studies suggest relatively low acute toxicity; however, long-term toxicity, repeated-dose toxicity, reproductive toxicity, immunological effects, and organ-specific safety profiles require systematic investigation. Particular attention should be given to hepatic metabolism because extensive biotransformation may influence both efficacy and toxicity. Furthermore, potential interactions with conventional anticancer drugs should be evaluated to determine whether β-ionone acts synergistically, antagonistically, or alters drug metabolism.

Mechanistic studies should be a focus in the future, including elucidating the precise molecular targets and signaling pathways, as well as interactions with pathways involved in cell survival, including the PI3K/Akt/mTOR, MAPK/ERK, and JAK/STAT3 pathways. The introduction of nanocarrier-based delivery systems may help address formulation challenges by facilitating tumor-targeted delivery of β-ionone, improving its solubility, and potentially enhancing its stability and bioavailability. Furthermore, combination therapy with β-ionone may enhance chemotherapeutic effects and sensitise resistant cells through its multimodal mechanism of action, which targets multiple apoptotic and survival pathways. Optimization of tumor-specific dosing schedules and the development of more bioactive β-ionone derivatives may further increase its translational potential.

## 10. Conclusions

β-Ionone is an emerging, potentially valuable natural apocarotenoid with anti-cancer activity in preclinical settings. Experimental evidence suggests that it can exert biological effects through mitochondrial apoptosis, modulation of Bax/Bcl-2 signalling, cytochrome c release, activation of caspases, oxidative stress, modulation of the cell cycle, and cancer-type-specific modulation of survival pathways. Recently, β-ionone has also been shown to mediate ferroptosis in gastric cancer models via the Wnt/β-catenin pathway. But the evidence is mainly preclinical and isn’t robust in all mechanisms and tumor types. While encouraging results have been obtained, numerous important research gaps remain to be addressed before the use of β-ionone in clinical applications is possible. First, in-depth mechanistic studies are needed to determine the direct molecular targets and to differentiate validated pathways from predicted interactions. Second, more detailed studies of pharmacokinetic and pharmacodynamic parameters, such as absorption, distribution, metabolism, elimination, bioavailability, and effective therapeutic levels, should be conducted. Third, there should be systematic toxicity studies with long-term safety evaluation and drug-to-drug interaction studies. Finally, formulation strategies such as nanodelivery and combination therapies require further optimisation to improve stability, tumour targeting, and therapeutic efficacy. Well-designed in vivo studies and validation in clinically relevant cancer models, pharmacological optimisation, and subsequent clinical evaluation will all be important in further research. The challenges addressed need to answer whether β-ionone and its analogues can serve as therapeutic drugs for cancer treatment.

## Data Availability

No new data were created or analyzed in this study. Data sharing does not apply to this article.

## Abbreviations

ADME, absorption, distribution, metabolism, and excretion; AKT, protein kinase B; Bax, Bcl-2-associated X protein; Bcl-2, B-cell lymphoma 2; CCDs, carotenoid cleavage dioxygenases; CDK4, cyclin-dependent kinase 4; CRC, colorectal cancer; DR5, death receptor 5; EPR, enhanced permeability and retention; ERK, extracellular signal-regulated kinase; FADD, Fas-associated protein with death domain; GPX4, glutathione peroxidase 4; JAK, Janus kinase; MAPK, mitogen-activated protein kinase; MMP, mitochondrial membrane potential; MOMP, mitochondrial outer membrane permeabilization; mTOR, mechanistic target of rapamycin; NF-κB, nuclear factor kappa B; OR51E2, olfactory receptor family 51 subfamily E member 2; PI3K, phosphatidylinositol 3-kinase; ROS, reactive oxygen species; SAR, structure–activity relationship; STAT3, signal transducer and activator of transcription 3; TRAIL, tumor necrosis factor-related apoptosis-inducing ligand; Wnt, wingless-related integration site.
